# KGAP: An RDF knowledge graph of agricultural commodity prices

**DOI:** 10.1016/j.dib.2026.112607

**Published:** 2026-02-19

**Authors:** Filipi Miranda Soares, Luís Ferreira Pires, Fernando Elias Corrêa, Luiz Olavo Bonino da Silva Santos, Kelly Rosa Braghetto, Dilvan de Abreu Moreira, Debora Pignatari Drucker, Alexandre Cláudio Botazzo Delbem, Antonio Mauro Saraiva

**Affiliations:** a Faculty of Electrical Engineering, Mathematics and Computer Science, University of Twente, Drienerlolaan 5, Enschede, 7522 NB, Overijssel, the Netherlands; b Polytechnic School, University of São Paulo, Av. Prof. Luciano Gualberto, 158, Butantã, São Paulo, 05508-010, SP, Brazil; c MISTEA, University of Montpellier, INRAE & Institut Agro, 2 place Pierre Viala, Montpellier Cedex 2, 34060, France; d Luiz de Queiroz College of Agriculture, University of São Paulo, Center for Advanced Studies on Applied Economics, Av. Pádua Dias, 11, Piracicaba, 13400-970, SP, Brazil; e Leiden University Medical Center, Human Genetics, Albinusdreef 2, Leiden, 2333 ZC, South Holland, the Netherlands; f Institute of Mathematics and Statistics, University of São Paulo, Rua do Matão, 1010, São Paulo, 05508-090, SP, Brazil; g Institute of Mathematics and Computer Science, University of São Paulo, Av. Trabalhador São-carlense, 400, São Carlos, 13566-590, SP, Brazil; h Embrapa Digital Agriculture, Av. André Tosello, 209, Campinas, 13083-886, SP, Brazil

**Keywords:** Agricultural economics, Semantic web, SPARQL, Agricultural products, Price, Time series, RDF

## Abstract

This article presents the Knowledge Graph for Agricultural Prices (KGAP), which is a knowledge graph (KG) that integrates agricultural commodity prices data from three major Brazilian institutions: Cepea, Conab, and Ipea. The datasets, originally published in heterogeneous formats, were harmonized and converted into RDF/Turtle using the Almes Core metadata schema as the data model. Agricultural products were classified with the Agricultural Product Types Ontology (APTO), and geographic references were aligned with GeoNames identifiers, ensuring semantic consistency and adherence to the FAIR data principles. KGAP is archived on Zenodo and GitHub, and hosted on the Platform Linked Data Nederland (PLDN) with a public SPARQL endpoint. It contains metadata, price observations, product types, and location entities, allowing users to query and compare agricultural prices across institutions, regions, and time periods. The knowledge graph can potentially support applications in agricultural economics, policy analysis, journalism, data science, and machine learning. By explicitly modeling metadata such as reference quantities, KGAP enables semantically-aware queries that prevent common analytical errors and reveal insights previously obscured by data heterogeneity.

Specifications Table

**Table:** 

Subject	Computer Science
Specific subject area	Agricultural price time series, semantic interoperability, and knowledge graph engineering.
Type of data	- Original tabular data in XLS. - Harmonized data in CSV. - RDF graphs in Turtle (.ttl).
Data collection	Agricultural price datasets were retrieved from the public portals of Cepea, Conab, and Ipea. From Cepea, time series data were collected from their inception up to the cut-off date of June 2023, covering crystal sugar (multiple types), fed cattle, Arabica and Robusta coffee, and soybean. From Conab, price indices were obtained for fed cattle (weekly data from January to August 2023) and for sugar, coffee, and soybean (monthly data from January to December 2023). From Ipea, time series spanning their inception to June 2023 were gathered for leather, cellulose, paper, tobacco, and wood products.
Data source location	Original data - Cepea: Center for Advanced Studies on Applied Economics (Cepea), Luiz de Queiroz College of Agriculture, University of São Paulo, Piracicaba, Brazil (GeoNames ID: 6324347) **Original data - Conab**: National Supply Company (CONAB), Ministry of Agriculture, Brasília, Federal District, Brazil (GeoNames ID: 3469058) **Original data - Ipea**: IpeaData, Institute for Applied Economic Research (Ipea), Ministry of Planning and Budget, Brasilia, Federal District, Brazil (GeoNames ID: 3469058)
Data accessibility	Raw data (Zenodo): https://doi.org/10.5281/zenodo.12163228, https://doi.org/10.5281/zenodo.12170310, https://doi.org/10.5281/zenodo.12169699 Harmonized data (Zenodo): https://doi.org/10.5281/zenodo.12580972 RDF graphs: https://doi.org/10.5281/zenodo.13741165, https://data.pldn.nl/FilipiSoares/AgriPrices-1/ APTO (AgroPortal and GitHub): https://agroportal.lirmm.fr/ontologies/APTO, https://github.com/AlmesCore/APTO/tree/main.

## 1 Value of the Data

• FAIR and interoperable agricultural data: The Knowledge Graph for Agricultural Prices (KGAP) provides the first FAIR-compliant and semantically enriched representation of Brazilian agricultural price index data, integrating information from Cepea, Conab, and Ipea.
• Supports multiple user communities: Researchers, journalists, policymakers, and farmers can directly access harmonized and queryable agricultural price data to support analysis, media reporting, and evidence-based decision-making.
• Reusable semantic framework: The data model and metadata align with the Almes Core metadata schema, the APTO ontology, and GeoNames, enabling reuse by other projects working with agricultural, economic, or geographic datasets.
• Machine-readable and queryable access: Data are published as Linked Open Data and available through a public SPARQL endpoint, allowing users to perform flexible and reproducible analyses.
• Contributes to open government and transparency: Developed under Brazils 5th National Action Plan on Open Government [1], KGAP demonstrates a scalable approach for improving public data interoperability in agriculture.
• The dataset provides semantically harmonized, multi-source agricultural price time series that are potentially suitable for training, validating, and benchmarking machine learning and statistical learning models, including neural networks, Gaussian process regression, and ensemble forecasting methods.
• The explicit representation of products, locations, time, and provenance can potentially facilitate feature extraction, data integration, and reproducibility in data-driven price forecasting, risk assessment, and policy analysis workflows.

## 2 Background

Agricultural price index data play a critical role in shaping public policy and informing market decisions. The most common analytical applications include time-series modeling and forecasting, which support the evaluation of market dynamics and volatility (e.g., [2], [3], [4], [5]), as well as spatial or regional price comparisons that reveal disparities in production costs, market integration, and food accessibility (e.g., [6], [7], [8], [9]). These analytical approaches are widely used in agricultural economics to inform subsidy design, food security monitoring, and supply-chain regulation. A broad body of empirical research, both in Brazil and internationally, has demonstrated how price integration metrics, regional price differentials, and transmission models contribute to understanding commodity price transmission, inflationary pressures, and long-term structural trends in agricultural markets.

Recent literature in agricultural and commodity economics has increasingly adopted machine learning and advanced statistical learning techniques to analyze and forecast price dynamics characterized by nonlinearity, volatility, seasonality, and sensitivity to external shocks. Neural networkbased models and related approaches have been widely explored for commodity price and index forecasting due to their ability to capture complex temporal dependencies [2], [3]. Gaussian process regression has also gained attention for commodity price analysis, offering flexible nonparametric modeling with explicit representations of predictive uncertainty [10], [11]. In addition, ensemble and composite forecasting strategies that integrate multiple models or data sources have been shown to improve robustness in agricultural and financial time-series analysis [4], [5], [12]. Collectively, these developments highlight the growing demand for harmonized, high-quality price datasets capable of supporting data-driven modeling, benchmarking, and validation workflows.

The fragmentation of data sources not only undermines the potential for cross-institutional integration and machine-learning applications, but also prevents full alignment with the FAIR data principles, particularly principle I (interoperability) [13]. This challenge was recognized in Brazils 5th National Action Plan on Open Government [1], which emphasized the need for interoperable public data in strategic sectors, including agriculture. In response, a multi-institutional team led by the GO FAIR Agro Brazil Network^1^ and key government agencies initiated the development of a unified framework for publishing agricultural price index data. KGAP is the outcome of that initiative.

## 3 Data Description

**Fig. 1:**
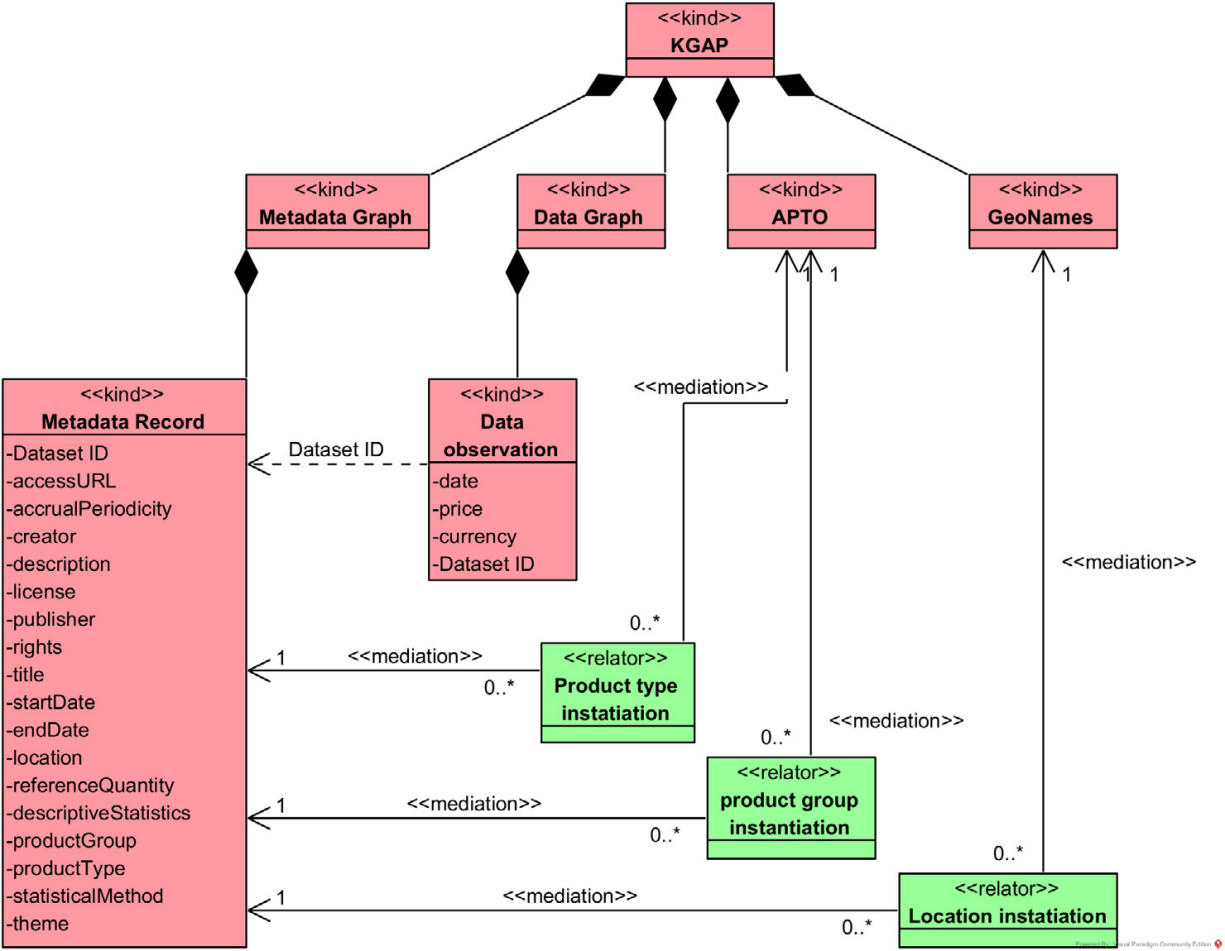
KGAP OntoUML Conceptual Model.

The conceptual model presented in Fig. 1 represents KGAP structure, which is based on the Almes Core metadata schema [14]. It contains four main graphs: Metadata Graph, Data Graph, APTO Graph, and GeoNames Graph. These four graphs were generated in RDF/Turtle and archived on Zenodo [9]. The conceptual model was specified using the OntoUML modeling language [15], [16], ensuring ontological rigor and semantic clarity in the representation of KGAP components and their relationships.

Starting with the Metadata Graph, it includes a description for each dataset, or more specifically, their metadata records. Each record contains the attributes displayed in Fig. 1. The list of metadata fields and their description is shown in Table A.1. Each dataset is identified as a dcat:Dataset, and unique URIs for each dataset were generated using hashes derived from files stored on GitHub.

The fields alm:productType and alm:productGroup in the Metadata Graph are instantiated with URIs of classes from APTO, implying that the values for these fields are URIs from this ontology. Similarly, sdo:location in the Metadata Graph is instantiated with GeoNames URIs. A subset of the GeoNames ontology was extracted to represent all Brazilian regions (i.e., states and cities) referenced in the metadata records of the Metadata Graph under the location field.

For the Data Graph, each data point includes date, price, and currency, following the Almes Core recommendations [14] as shown in Table 1.

**Table 1:** Data Graph Elements.

Term	Data Type	Definition

dc:date	date	A point or period of time associated with an event in the lifecycle of the resource. Date may be used to express temporal information at any level of granularity. Recommended practice is to express the date, date/time, or period of time according to ISO 8601-1 [ISO 8601-1] or a published profile of the ISO standard, such as the W3C Note on Date and Time Formats [W3CDTF] or the Extended Date/Time Format Specification [EDTF]. In case the date is a range, sdo:startDate and sdo:endDate should be used instead to indicate the beginning and the end of the period.
sdo:price	float	The offer price of a product.
sdo:currency	string	The currency in which the monetary amount is expressed.

We modeled these data points as RDF blank nodes of a Dataset ID from the Metadata Graph, as illustrated in the example below:



For data that were published as an interval, such as Conab’s Weekly Prices, the structure was adapted to use sdo:startDate and sdo:endDate instead of sdo:date.

## 4 Experimental Design, Materials and Methods

We developed KGAP in accordance with the core principles of Linked Data and Semantic Web, as originally articulated by Berners-Lee [17] and formalized through W3C standards such as RDF, RDFS, OWL, and validated through SPARQL queries [18], [19]. These principles comprise:

• Use of dereferenceable URIs to uniquely identify resources.
• Modeling of knowledge as RDF triples using well-defined vocabularies.
• Interlinking of data from heterogeneous sources.
• Enabling both human and machine interpretation of data semantics.

KGAP design followed a modular and layered architecture, separating metadata (descriptive information about datasets) from observation data (price variables over time), and aligning these layers to domain-specific and external ontologies (e.g., APTO, GeoNames).

Our approach draws from methodologies in Knowledge Graph Engineering, which is an emerging discipline that proposes systematic processes for KG construction, typically involving stages such as requirement analysis, modeling, implementation, and maintenance [20], [21].

We organized the Methods section according to the knowledge graph lifecycle stages (creation, hosting, curation, deployment) described by [22]. Their work outlines the processes required for building and maintaining knowledge graphs, encompassing stages such as creation, hosting, curation, and deployment.

### 4.1 Knowledge creation

In the Knowledge Creation stage, RDF triples are generated from raw data sources and linked with ontologies or external vocabularies. Creation can be manual or (semi-)automated, often using mappings or custom transformation logic [22]. In our case study, the knowledge creation was semi-automated, and followed the steps below:

1. Agricultural price datasets were extracted from three key Brazilian public institutions: Cepea (Dataset [1]), Conab (Dataset [2]), and Ipea (Dataset [3]). Each institution employed different publishing practices, formats, and metadata structures, requiring individual preprocessing strategies.
2. Raw data was cleaned and normalized using custom Python scripts (scripts [4] and [5]). In this process, we reconciled inconsistent column names, resolved character encoding issues, standardized date formats and measurement units, and enriched records with missing values, in accordance with predefined data and metadata templates. The resulting intermediate, preprocessed datasets are also archived on Zenodo [6].
3. Metadata was mapped to the Almes Core schema, which was developed in the early stages of this project and described in [23]. This mapping was achieved using the data_converter Python script that uses the RDFLib library, and is archived on Zenodo [7].
4. Metadata records were instantiated as dcat:Dataset and populated with properties from the Almes Core schema, capturing dataset-level attributes such as title, description, update frequency, temporal coverage, and provenance. In KGAP, each record corresponds to a dataset isolated from source aggregates by splitting on unique combinations of *location, product type*, and *publisher*. Any new combination yields a distinct dataset. For example, cane-sugar prices for São Paulo published by Cepea constitute a different dataset from those published by Conab.
5. Agricultural products in the metadata records were mapped to classes from APTO, also developed in an earlier stage of this project to model agricultural product types and documented in two previous publications [24], [25]. Geographical locations were aligned with GeoNames URIs for standardized spatial referencing.
6. Individual price observations were modeled as blank nodes and connected to their respective dataset entries using the alm:hasObservation property.

The creation of each graph described in Section 2 required specific pipelines, which are described in the sequel.

#### 4.1.1 Metadata graph creation

**Fig. 2:**
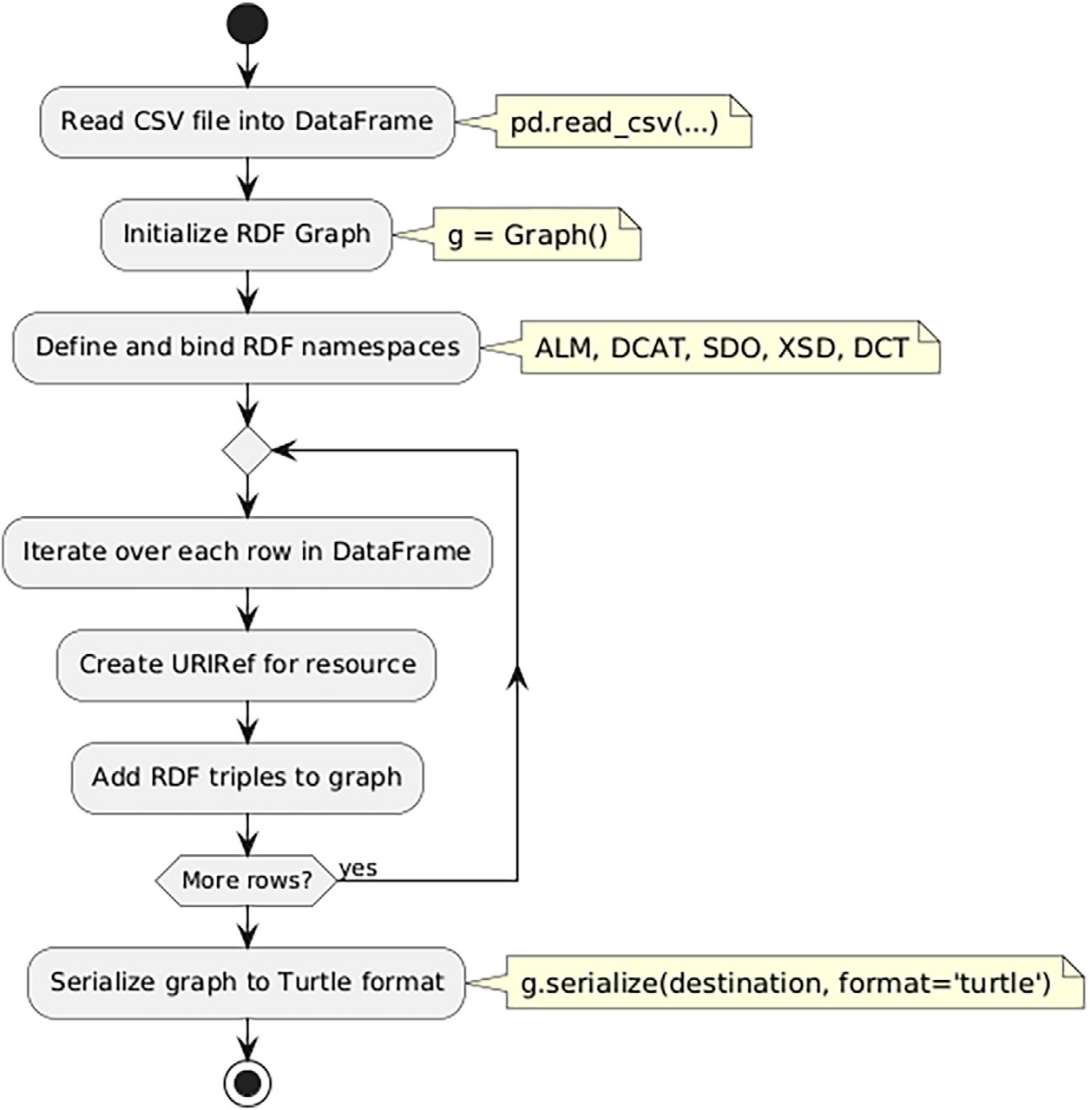
Metadata Converter Pipeline.

We designed a Python script (named metadata_converter.py and archived on Zenodo [7]) to convert the metadata CSV file previously generated (Zenodo [6]) into an RDF/Turtle graph. The script workflow is shown in Fig. 2.

The script begins by reading a CSV file containing metadata using the pandas library (df = pd.read_csv). Each row in this CSV file contains the metadata description of a dataset, corresponding to a time series for a specific product type, published by Cepea, Ipea, or Conab. Next, it initializes an RDF graph (g = Graph()) using rdflib. This graph stores the triples (subject-predicate-object relationships) for each dataset. To ensure semantic consistency, various namespaces are defined and bound to the graph (ALM, DCAT, SDO, XSD, DCT).

The script then iterates through each row in the CSV file as follows:

• Each resource is assigned a provisional URI. These URIs serve as temporary identifiers during the RDF generation process and are later replaced with hash-based URIs before publication.
• A Uniform Resource Identifier Reference URIRef is generated for the resource, which acts as the subject of the RDF triples.
• Several RDF triples are added to the graph, mapping the resource’s properties to RDF predicates, such as dct:title, dct:description, sdo:startDate, etc. Literal values such as dates and strings are converted using the appropriate datatype (e.g., XSD.date for dates and XSD.string for strings).

After all triples were added, the resulting graph was serialized in Turtle format and published on GitHub [8]. Listing 1 presents an example metadata record from this graph.

**Listing 1:**
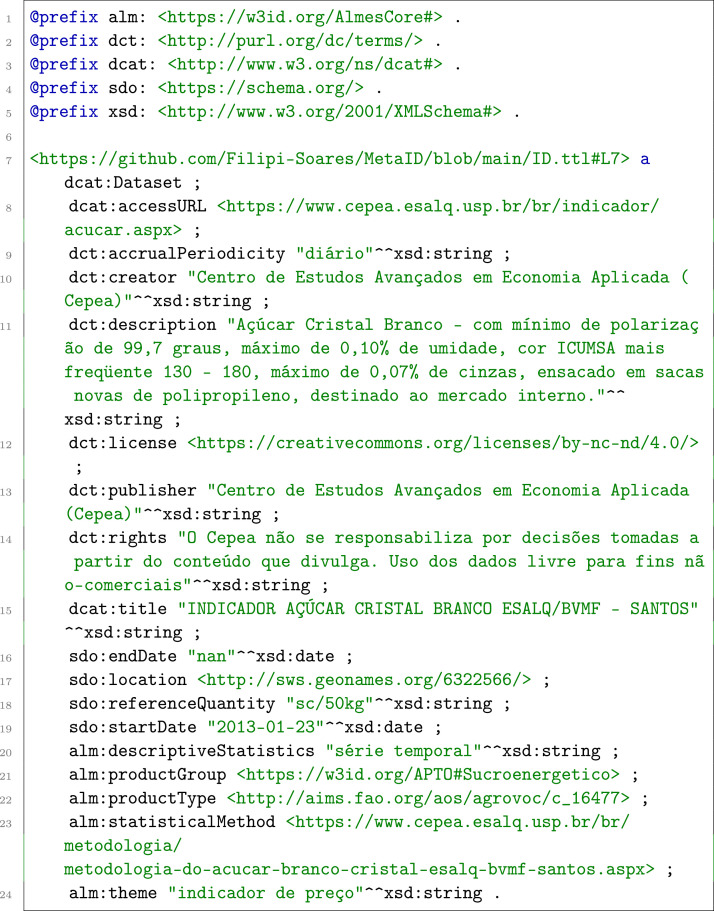
KGAP Metadata Example (Cepea Cane Sugar, São Paulo).

#### 4.1.2 GeoNames graph creation

As mentioned in Section 2, the sdo:location field in the Metadata Graph was populated with GeoNames URIs [26]. To ensure that users querying KGAP can view the location names associated with each URI, we extracted classes from the GeoNames ontology via their API. This extraction was limited to the specific locations included in the Metadata Graph, rather than the entire GeoNames ontology, and did not include the location hierarchy.

**Fig. 3:**
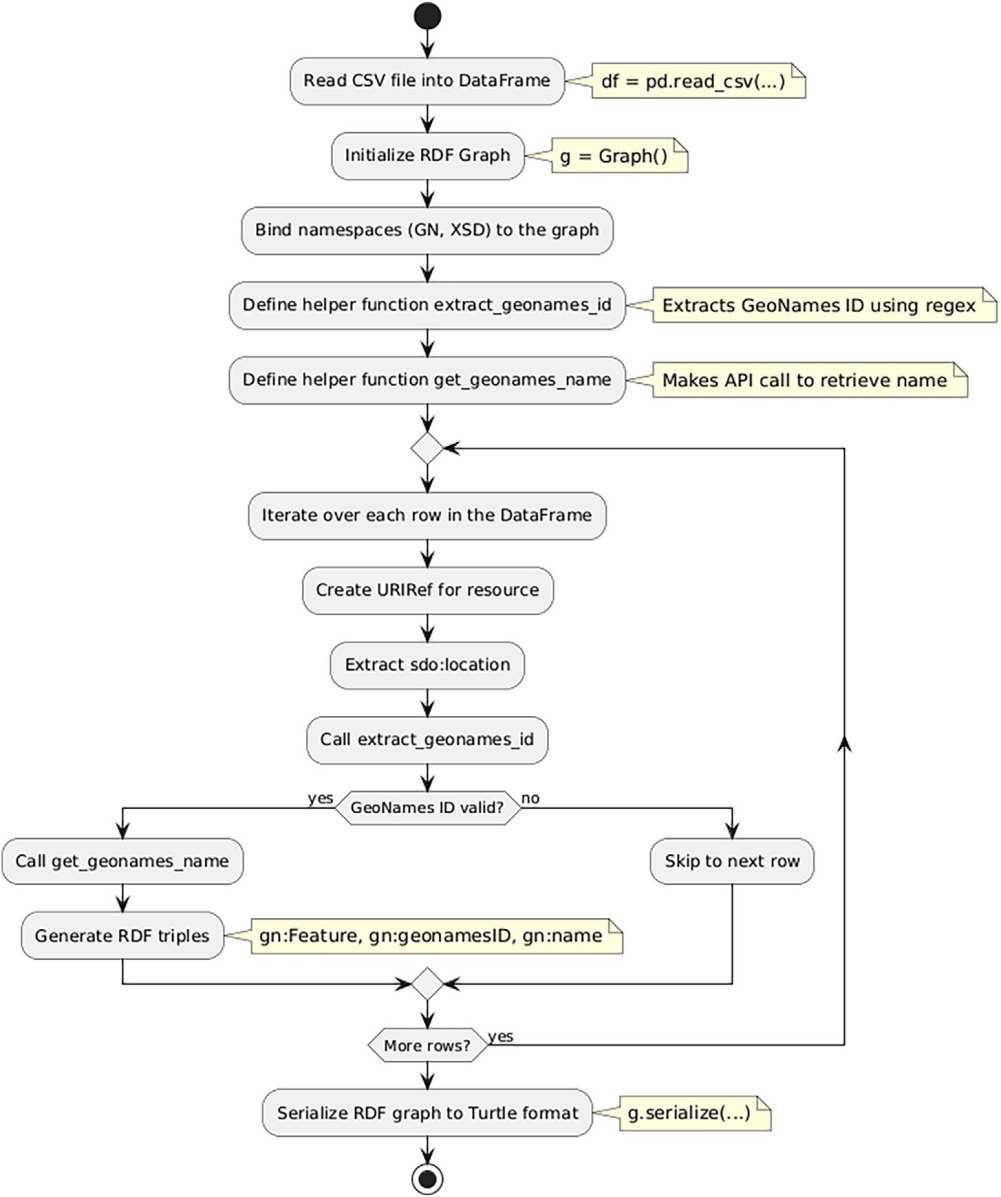
Geonames Converter Pipeline.

To achieve this, we developed a Python script (GeoNames.py, Zenodo [7]), which functions as shown in Fig. 3. This script extracts GeoNames information for the geographic locations provided in the dataset, enriches the data with GeoNames metadata, and converts it into RDF/Turtle for integration into KGAP.

The script begins by reading a CSV file into a pandas DataFrame, which contains the data from the Metadata Graph, including geographic location information. Next, an RDF graph is initialized to store RDF triples that are created when the script executes. To support semantic representation, the namespaces GN (representing GeoNames ontology) and XSD (for datatypes) are defined and bound to the graph using g.bind.

The script defines a helper function called extract_geonames_id, which uses regular expressions to extract the GeoNames ID from the location field in each row. This function identifies numerical IDs embedded within URL patterns that point to GeoNames resources.

Another helper function called get_geonames_name takes the extracted GeoNames ID and sends an API request to the GeoNames service using the requests.get function. If the request is successful, it parses the JSON response to extract the geographic feature name.

The script then iterates over each row in the dataset. For every row, a URIRef is created to represent the described resource. The script extracts the sdo:location field and calls extract_geonames_id to retrieve the GeoNames ID.

If a valid ID is found, the script uses get_geonames_name to fetch the geographic name and then constructs RDF triples to represent the geographic feature.

For each valid GeoNames ID, the script generates the following RDF triples:

• The geographic feature is typed as a gn:Feature.
• The GeoNames ID is linked to the resource using gn:geonamesID.
• The name of the geographic feature is linked using gn:name.

Finally, the graph is serialized to a Turtle file. An example is shown in Listing 2.

**Listing 2:**
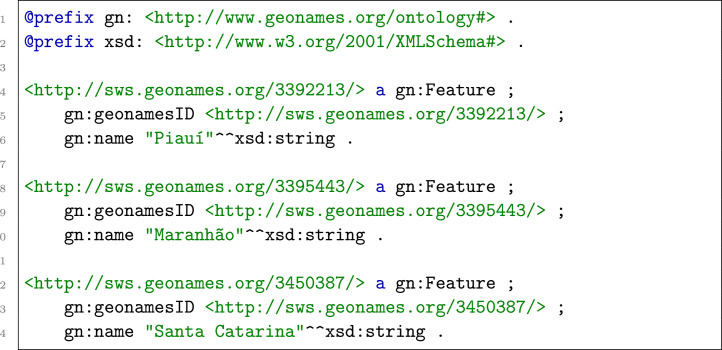
KGAP GeoNames Graph Example.

#### 4.1.3 APTO Graph creation

APTO was developed and validated in a prior stage of this project, as described in [24], [25], and it was developed by following the SABIO methodology [27]. APTO classes were used to populate the fields alm:productType and alm:productGroup in the Metadata Graph, enabling machine-readable classification of each datasets product scope.

The ontology source is hosted on GitHub^2^, while the W3ID namespace^3^ is configured with redirect rules to AgroPortal^4^, which handles content negotiation and ensures persistent accessibility and version control [28]. The specific version of APTO used in KGAP was also archived on Zenodo [9].

#### 4.1.4 Data graph creation

**Fig. 4:**
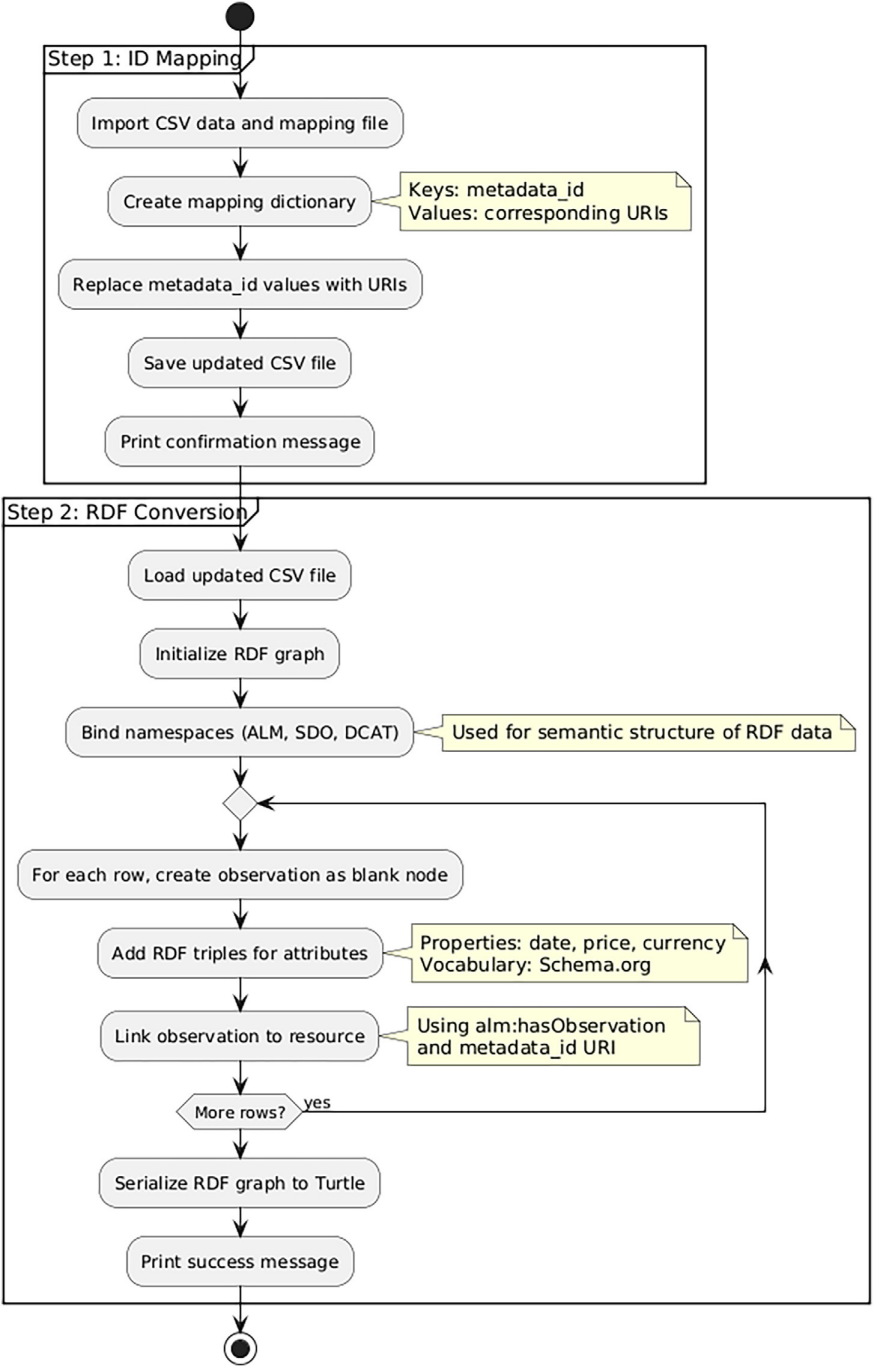
Data Converter Pipeline.

The dataset was initially provided in CSV format (see Zenodo [6]) and contained four fields: metadata_id, date, price, and currency. To transform this data into RDF, we developed a Python-based pipeline, represented in Fig. 4.

The pipeline is divided into two main steps. First, a script replaces the ID values^5^ in the metadata_id column with the corresponding URIs previously generated for the resources in the Metadata Knowledge Graph. The script loads the main dataset and the mapping file, builds a dictionary of ID-to-URI correspondences, replaces the original identifiers in the dataset, and saves the updated file for the next stage.

The second step focuses on transforming each row of the updated dataset into RDF. A new script loads the enriched CSV and initializes an RDF graph using the rdflib library. Namespaces from ALM, SDO, and DCAT are defined and bound to the graph to ensure consistent use of vocabularies.

Each row in the dataset is processed as an individual observation. For every observation, a blank node is created and annotated with RDF triples that describe its attributes (date, price, and currency). These values are linked using Schema.org predicates and typed according to the appropriate XML Schema datatypes (e.g., xsd:date, xsd:float, xsd:string).

To complete the model, each observation is linked back to its related metadata resource using the alm:hasObservation property and the URI from the metadata_id field. The final RDF graph is then serialized into Turtle format and saved to a file, ready for integration into the broader Knowledge Graph. Both scripts are implemented in a single Python notebook [7].

For daily price series, we record the reference day with sdo:date (Listing 3). For interval-based publications (e.g., weekly or monthly), we represent the coverage with sdo:startDate and sdo:endDate (Listing 4). All dates use the ISO 8601 format (YYYY-MM-DD).

**Listing 3:**
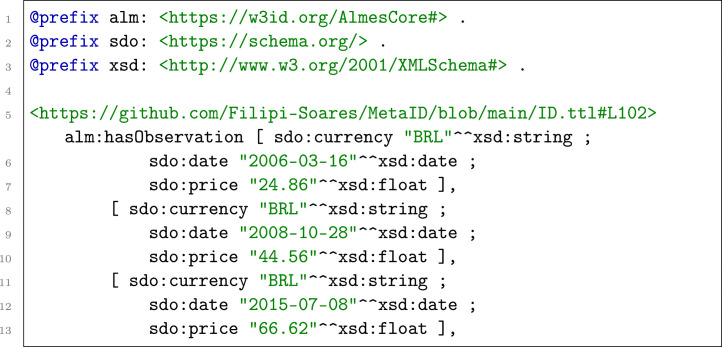
KGAP Data Graph. Example of Daily Prices.

**Listing 4:**
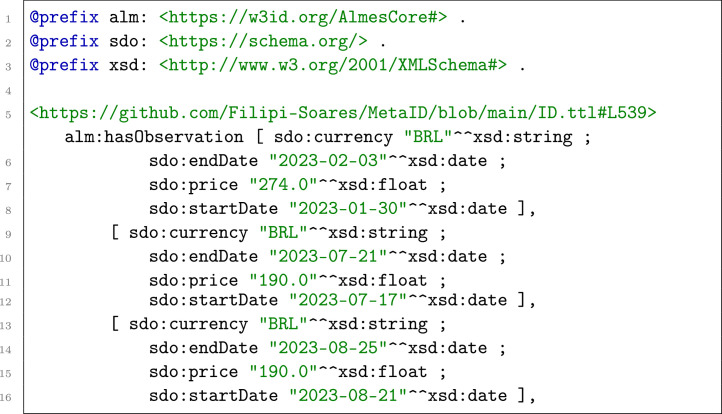
KGAP Data Graph. Example of Weekly Prices.

### 4.2 Knowledge hosting

Knowledge Hosting refers to the storage, publication, and long-term accessibility of a KG, typically through repositories like Linked Data platforms, or triple stores that support querying, versioning, and interoperability [22]. KGAP is hosted on the Platform Linked Data Netherlands (PLDN^6^), which provides robust infrastructure for storing RDF data as a triple store. In addition, KGAP has been archived on Zenodo [9].

### 4.3 Knowledge curation

According to [22], Knowledge Curation encompasses activities such as data cleaning, enrichment, assessment, and validation, to ensure the accuracy, completeness, and semantic richness of the KG. For KGAP, we carried out:

• Cleaning: We cleaned the input data to correct malformed entries (e.g., date formats like yyyy.mm) and resolve identifier mismatches (e.g., merged cells in the Conab data).
• Assessment: Internal tests ensured that the metadata conformed to Almes Core and that price entries were consistently typed and linked.
• Enrichment:
  • GeoNames enrichment was implemented by calling the GeoNames API for each location and appending gn:name and gn:geonamesID triples.
  • Semantic enrichment of products was achieved by mapping terms to APTO classes using string matching and manual curation.

### 4.4 Knowledge deployment

Knowledge Deployment involves making the KG usable for end applications, such as search, question answering, analytics, or decision support systems [22]. KGAP is queryable via a SPARQL endpoint^7^, and allows answering analytical questions such as the ones presented in Section 1.

Beyond the current semantic annotations provided by APTO and GeoNames, KGAP could be further extended through feature-extraction pipelines that integrate external economic, environmental, and social data sources to derive machine-learning-ready indicators (such as moving averages, volatility measures, or sentiment scores) for downstream analytical applications, as explored in knowledge-graph-driven machine learning research [29].

### 4.5 Query examples

KGAP supports a variety of analytical use cases that can be expressed through competency questions such as:

• What was the daily market price of a specific agricultural product (e.g., coffee beans) in a specific region (e.g., Minas Gerais)?
• How did the average monthly price of a commodity (e.g., fed cattle) evolve over a year?
• How do price reports from different institutions (e.g., Cepea and Conab) compare for the same product and time window?
• How do agricultural prices differ between major producing states such as Paraná and Mato Grosso?
• How do differences in packaging size (e.g., 30kg vs. 50kg) affect price comparisons between sources?
• Which products showed the greatest price volatility over a specific period and region?

To validate the technical structure of KGAP, we designed a set of SPARQL queries derived from the defined competency questions. All queries are publicly available through a dedicated URI on PLDN and are archived on Zenodo [9]. A more detailed discussion of query design and debugging is provided in Chapter 10 of [30].

As KGAP is hosted on PLDN, all queries were executed using the platforms Virtuoso SPARQL endpoint, allowing results to be reproduced by any user who access the public endpoint. Accordingly, all visualizations presented in this paper were generated using query results obtained through the PLDN Virtuoso SPARQL endpoint. We discuss some query examples in the sequel.

#### 4.5.1 Query 1: Price of a product on a specific date and location

One common type of query users perform to agricultural price index datasets is retrieving the price of a specific commodity on a given day in a particular location in Brazil. To simulate this use case, we generated a query to retrieve the price of ‘Café em grãos’ (coffee beans) for the Brazilian State of Minas Gerais, on 2023-06-01. The query results are shown in Table 2. This full query is accessible through a URI^8^.

**Table 2:** Price of Coffee Beans in Minas Gerais on June 1, 2023.

Location	Product Type	Date	Price	Currency
Minas Gerais	http://aims.fao.org/aos/agrovoc/c_28379	2023-06-01	893.82	BRL

#### 4.5.2 Query 2: Time series data visualization

**Fig. 5:**
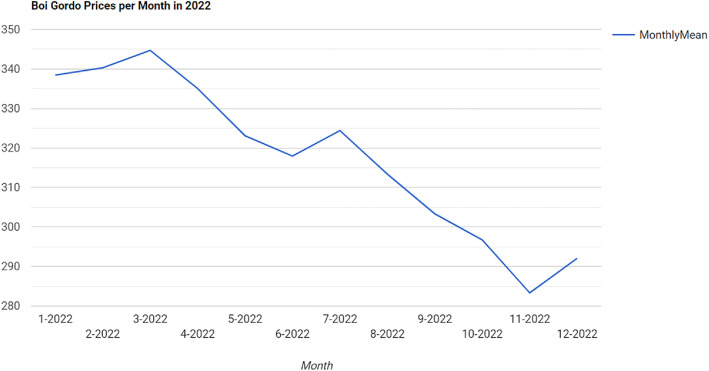
Query Results Showing the Mean Price for ’Boi Gordo’ in 2022.

We assume a user wants to see the evolution of the price of ‘Boi Gordo’ (Fed cattle, in English) across the year 2022 based on the prices published by Cepea. These prices are published on a daily but irregular basis, since Cepea does not publish price index data on weekends and holidays. The goal is to calculate the mean price for each month of 2022 and plot a chart with the results. The query result is shown in Fig. 5. The full query, its results, and the visualization are accessible through a URI^9^.

In addition to visualization, this query produces a temporally ordered price series that can be exported in tabular form (e.g., CSV) and used as input for downstream analytical or predictive modeling workflows. Such outputs are compatible with standard data-science pipelines and machine-learning libraries (e.g., pandas, scikit-learn, TensorFlow), and may support tasks such as forecasting, trend analysis, or anomaly detection without requiring substantial additional data preparation.

#### 4.5.3 Query 3: Comparing prices from two organizations

The goal of this query was to compare the monthly prices of Cane Sugar (‘Açúcar Cristal’) from Cepea and Conab, for the first semester of 2023, in the State of São Paulo. Cepea publishes prices on a daily basis, whereas Conab’s prices are typically monthly or weekly. The query aggregates the daily prices from Cepea to calculate monthly averages and compares them to Conab’s monthly prices for the same time period.

This query contains two subqueries, each designed to retrieve and calculate monthly prices for Cepea and Conab, respectively. The results from the two subqueries are then combined to compare the prices from both institutions:

• The first subquery retrieves daily prices for Cane Sugar published by Cepea and aggregates them to produce monthly mean prices.
• The second subquery retrieves the monthly prices for Cane Sugar published by Conab. This subquery works similarly to the first but assumes that Conab publishes data on a monthly basis rather than daily. The monthly mean prices for Cepea and Conab are calculated in their respective subqueries, while the outer query combines the results to provide a side-by-side comparison of prices for each month.

**Fig. 6:**
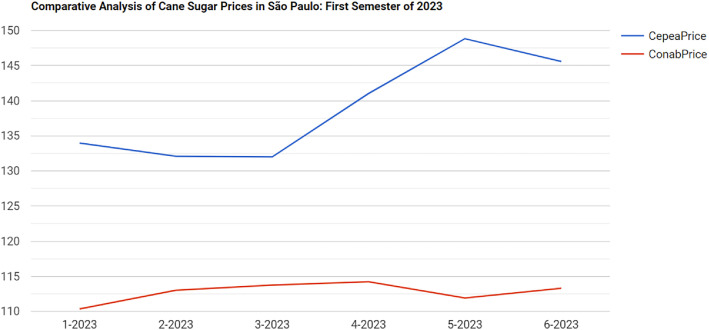
Query results showing prices from Cepea and Conab for cane sugar for the first semester of 2023.

The query orders the results chronologically by month. The results were used to plot the chart shown in Fig. 6, which compares prices from both institutions. This query and its results are accessible through the URI.^10^

By aligning prices from different institutions and normalizing reference quantities, this query performs a key preprocessing step required for machine learning applications, namely the generation of harmonized features from heterogeneous data sources.

#### Changes Introduced in the Second Subquery of Query 3 for Adjusting Prices Per Kg

**Fig. 7:**
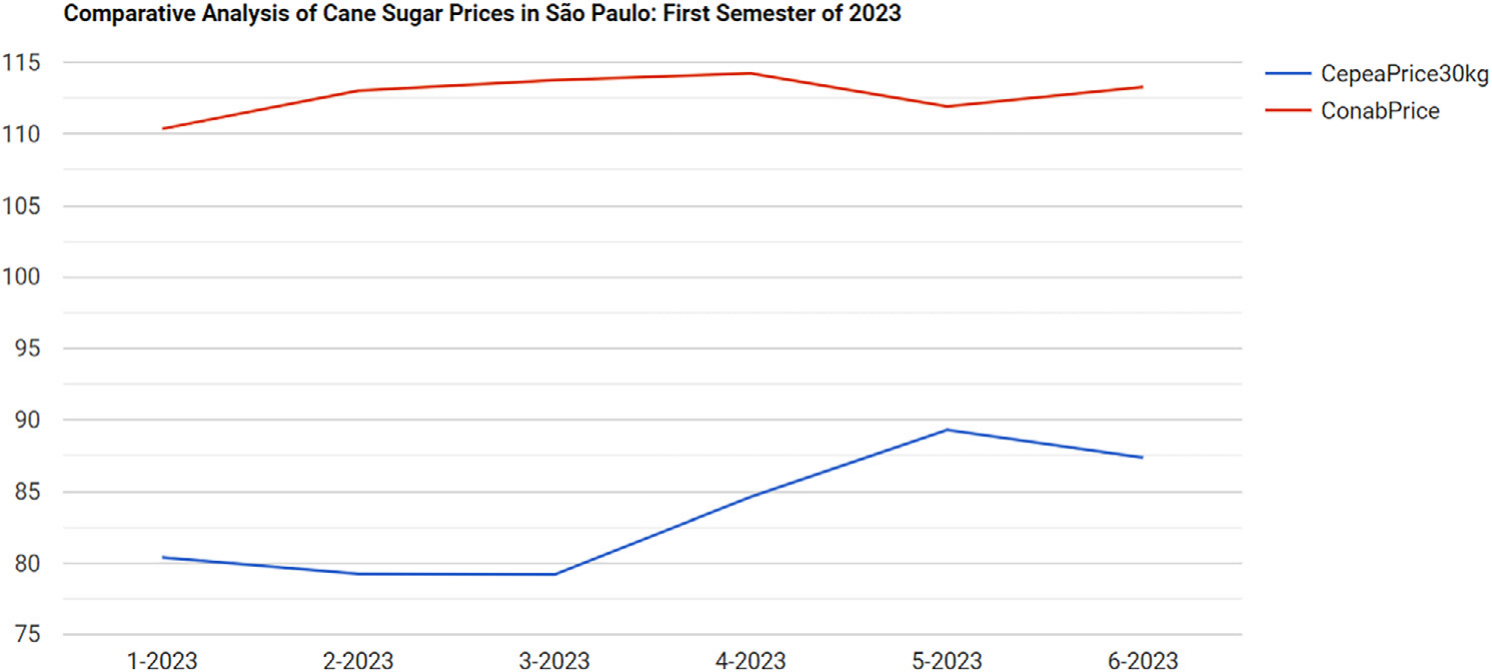
Harmonized query results showing prices from Cepea and Conab for cane sugar for the first semester of 2023.

Fig. 6 shows that cane sugar prices reported by Cepea were higher than those from Conab during the first semester of 2023. However, this initial comparison requires caution: Cepea’s prices are based on a 50kg bag of sugar (as defined in sdo:referenceQuantity), while Conab’s prices are based on a 30kg bag, so these values are not directly comparable. This difference can be addressed by adjusting the SPARQL query to account for the different reference quantities. The key change in this second version is the normalization of Cepea’s prices to match the 30kg bag weight used by Conab, to ensure that the price comparison reflects the same product quantity. As shown in Fig. 7, the normalized analysis reveals that Cepea’s sugar prices are actually lower than Conab’s – the opposite of what was shown in Fig. 6.

This discrepancy can be due to the principle of economies of scale. In Brazil, as in many markets, purchasing a larger quantity of a product, such as, e.g., a 50kg bag of sugar, often results in a lower price per unit of weight due to bulk discounts. Sellers reduce the price per kilogram as the order size increases, which reflects operational savings in packaging, logistics, and transaction costs. Therefore, Cepeas lower per-kilo price, once adjusted for bag size, aligns with this market behavior. This query and its results are accessible via a URI.^11^

#### 4.5.4 Query 4: Comparing prices between two locations

**Fig. 8:**
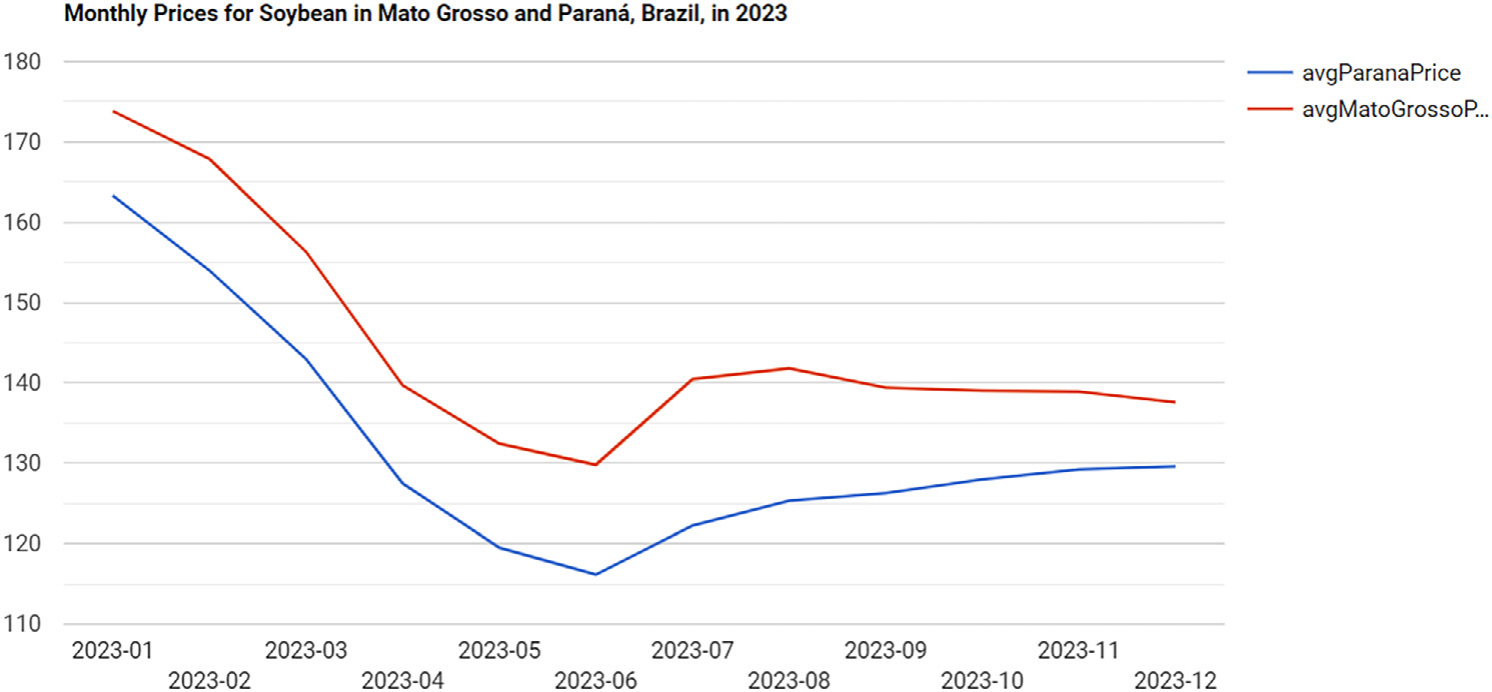
Prices for Soybean in Paraná and Mato Grosso in 2023.

As done in [31], price index data analysis often involves comparing prices from two or more regions in Brazil. In KGAP, this type of analysis can also be performed with a SPARQL query. We used the Conab data as starting point. Suppose someone then wants to compare the prices of soybeans in 2023 between Paraná and Mato Grosso states, the two most important soybean-producing states in Brazil. This query groups the results by month and year, displaying them in chronological order. The results are presented in a table format, from which the chart in Fig. 8 was generated. The chart indicates similar fluctuations in both regions for soybean prices throughout the year. Additionally, it shows that soybean prices in Paraná remained consistently lower than those in Mato Grosso over the entire year. This query and its results are accessible via a URI.^12^

Regionally dispersed price series such as these could support downstream analytical tasks, including the detection of abnormal price divergences or structural differences between markets, which are commonly explored using clustering or anomaly detection techniques [32].

### 4.6 Query reproduction

Although some of the queries presented in this paper involve nested structures and blank nodes, the full set of queries archived on Zenodo [9] can be reused as templates to support users who may be unfamiliar with SPARQL. These queries illustrate common analytical tasks and are intended as practical examples that can be adapted and simplified according to specific use cases.

Users more familiar with relational databases should be aware of the differences between SPARQL and SQL. While SPARQL and SQL differ syntactically and operate over distinct data models, many common analytical operations have direct equivalences in both languages. SQL SELECTFROMWHERE clauses correspond to SPARQL graph pattern matching, relational JOIN operations can be expressed through shared variables, and aggregation functions such as GROUP BY and AVG are supported in both query paradigms [33]. A key distinction is that SPARQL operates over explicit semantic relationships, allowing queries to traverse linked entities across datasets without requiring a predefined relational schema [33], [34]. This is especially relevant for integrating heterogeneous data sources, as in the case of KGAP.

## Limitations

The current version of KGAP has limitations that should be considered when assessing its scope and potential applications.

KGAP includes only the geographic locations explicitly referenced in the source datasets. Although locations are aligned with GeoNames identifiers, the absence of an explicit spatial hierarchy (e.g., municipalitystateregion relationships) limits more advanced spatial aggregation and multi-scale geographic analysis. Incorporating GeoNames hierarchical relations would enable richer spatial queries and broader regional analyses.

The coverage of agricultural products in KGAP is also limited. At present, the knowledge graph includes fed cattle, sugar, coffee, soybeans, and a small number of industrial products from Ipea. While this reflects the availability and relevance of the source data, it restricts the breadth of its potential analytical use cases.

From a technical perspective, although RDF provides a flexible and expressive data model, some analytical tasks are more complex to express in SPARQL than in comparable relational database systems. The use of nested structures and blank nodes requires multiple indirections to retrieve price observations, increasing query complexity and development effort. In addition, aligning datasets published at different temporal granularities (e.g., daily versus monthly) required string-based operations such as SUBSTR, which reduced query readability.

KGAP is currently constructed from batch-published datasets released periodically by the source institutions and does not support real-time data ingestion. As a result, KGAP is not suitable for real-time decision-making scenarios. Nevertheless, the modular separation between metadata, observation data, and semantic enrichment layers supports incremental updates and versioned data publication. Near-real-time ingestion pipelines and streaming extensions may be explored as the data providers publication practices evolve.

With respect to data quality, CEPEA and IpeaData provided sufficient documentation to complete the metadata template used to deploy the metadata graph. However, limitations were identified in the datasets published by CONAB, specially because they did not provide explicit titles for their datasets. To ensure consistency, artificial titles were generated by combining the publication frequency (e.g., average monthly prices or average weekly prices), product name, and commercialization level (e.g., wholesale (*atacado*, in Portuguese) or producer price (*produtor*, in Portuguese)).

CONAB datasets lacked descriptive metadata, explicit start dates for the price series, and references to the methodologies used for price calculation. Requests for this missing information were formally submitted to CONAB under Brazilian Law No. 12.527/2011, which guarantees public access to information, via the Gov.br portal. The responses received were incomplete, and as a result, several metadata fields remain unavailable. These gaps limit the interpretability and potential reuse of the affected datasets [30].

Beyond these data-quality constraints, this article does not include performance benchmarks, predictive modeling, or analytical validation experiments. Query execution time and scalability in RDF-based systems depend strongly on factors such as triple-store configuration, indexing strategies, query formulation, and deployment environment, making generic benchmarks difficult to interpret and reproduce. Likewise, assessing suitability for machine-learning applications would require task-specific feature engineering, model selection, and evaluation protocols. As a report on a specific dataset, this work focuses on describing the process of producing a FAIR, semantically harmonized dataset intended to support such evaluations in downstream analytical studies, rather than prescribing or optimizing particular computational workflows.

Despite these limitations, KGAPs RDF-based and modular architecture can support incremental updates and continuous data publication. Future extensions may explore near-real-time ingestion via API integration, provenance modeling (e.g., PROV-O) to capture uncertainty and update history, and tighter coupling with external analytical platforms, enabling online learning and uncertainty-aware analytics while preserving KGAPs role as domain-agnostic data infrastructure.

## Ethics Statement

The authors confirm that this work complies with the ethical requirements for publication in Data in Brief. The study did not involve human participants, animal experiments, or data collected from social media platforms. All datasets integrated in the Knowledge Graph for Agricultural Prices (KGAP) were obtained from publicly available sources (Cepea, Conab, and Ipea), and their use complies with the data redistribution and citation policies of these institutions.

## Data Availability

Data and codes used in this study are available as follows:

1. Soares, F., Corrêa, F. E., & Centro de Estudos Avançados em Economia Aplicada (CEPEA). (2024). Raw Data from Cepea on Sugar, Fed Cattle, Coffee, and Soybean Price Indexes [Data set]. Zenodo. https://doi.org/10.5281/zenodo.12163228
2. Soares, F., Corrêa, F. E., & Companhia Nacional de Abastecimento (Conab). (2024). Raw Price Index Data from Conab on Sugar, Fed Cattle, Coffee, and Soybean [Data set]. Zenodo. https://doi.org/10.5281/zenodo.12170310
3. Soares, F., Corrêa, F. E., & Instituto de Pesquisa Econômica Aplicada (Ipea). (2024). Raw Price Index Data from IpeaData on Leather and Leather Goods, Cellulose Pulp, Paper and Paper Products, Tobacco Products, and Wood Products [Data set]. Zenodo. https://doi.org/10.5281/zenodo.12169699
4. Soares, F. (2024). transform_dates.py script. Zenodo. https://doi.org/10.5281/zenodo.12532448
5. Soares, F. (2024). Excel Files Merger. Zenodo. https://doi.org/10.5281/zenodo.12206148
6. Soares, F. (2024). Treated Price Index Data from Cepea, Ipea, and Conab [Data set]. Zenodo. https://doi.org/10.5281/zenodo.12580972
7. Soares, F. M. (2024). Scripts for converting CSV data to RDF/Turtle using Python. [Computer software]. Zenodo. https://doi.org/10.5281/zenodo.13687647
8. Soares, F. M. (2024). Metadata file in Turtle. GitHub. https://github.com/Filipi-Soares/MetaID/blob/main/ID.ttl
9. Soares, F. M. (2024). The C4AI Knowledge Graph on Agricultural Prices (C4AI-KGAP) (v1.2 - This version includes a file with SPARQL queries.) [Dataset]. Zenodo. https://doi.org/10.5281/zenodo.13741165

## CRediT authorship contribution statement

**Filipi Miranda Soares:** Conceptualization, Methodology, Software, Formal analysis, Investigation, Data curation, Writing – original draft, Writing – review & editing, Visualization. **Luís Ferreira Pires:** Conceptualization, Writing – review & editing, Supervision. **Fernando Elias Corrêa:** Conceptualization, Methodology, Investigation, Resources, Writing – review & editing. **Luiz Olavo Bonino da Silva Santos:** Conceptualization, Writing – review & editing, Supervision. **Kelly Rosa Braghetto:** Conceptualization, Writing – review & editing. **Dilvan de Abreu Moreira:** Conceptualization, Validation, Writing – review & editing. **Debora Pignatari Drucker:** Conceptualization, Writing – review & editing. **Alexandre Cláudio Botazzo Delbem:** Writing – review & editing, Project administration, Funding acquisition. **Antonio Mauro Saraiva:** Conceptualization, Resources, Writing – review & editing, Supervision, Project administration, Funding acquisition.

## Figures and Tables

**Table A.1:** Metadata Graph Elements.

Term	Data Type	Definition
dcat:Dataset	(Class)	“A collection of data, published or curated by a single agent, and available for access or download in one or more representations” [35]
dct:identifier	anyURI	“An unambiguous reference to the resource within a given context” [36].
dcat:accessURL	anyURI	“A URL of the resource that gives access to a distribution of the dataset, e.g., landing page, feed, or SPARQL endpoint” [35].
dct:accrualPeriodicity	Literal	“The frequency with which items are added to a collection. Recommended practice is to use a value from the Collection Description Frequency Vocabulary (DCMI-COLLFREQ)” [36].
dc:creator	Literal	“An entity primarily responsible for making the resource” [36].
dct:description	Literal	“May include, but is not limited to: an abstract, a table of contents, a graphical representation, or a free-text account of the resource” [36].
dct:license	anyURI	“A legal document giving official permission to do something with the resource” [36].
dc:publisher	Literal	“An entity responsible for making the resource available” [36].
dc:rights	Literal	“Information about rights held in and over the resource” [36].
dc:title	Literal	“A name given to the resource” [36].
sdo:startDate	date	“The start date and time of the item (in ISO 8601 date format). In this application profile, it is used to inform the start date of a price index time series” [37].
sdo:endDate	date	“The end date and time of the item (in ISO 8601 date format)” [37]. In KGAP, this field in the metadata description is used to inform the end date of a price index time series. In case the price index is still active, this field was left blank.
sdo:location	anyURI	“The location of, for example, where an event is happening, where an organization is located, or where an action takes place” [37]. In KGAP, values were added from Geonames.org to represent the locations where the prices were observed.
sdo:referenceQuantity	Literal	The reference quantity for which a certain price applies, e.g. BRL per 50kg bag of sugar. This property serves as a replacement for unitOfMeasurement in advanced cases where the price does not relate to a standard unit. In KGAP, it indicates the reference quantity of the traded product.
alm:descriptiveStatistics	Literal	Summarizes and describes the main features of a dataset. When used in the context of a time series, it includes metrics such as mean, variance, and trends over time.
alm:productGroup	anyURI	A ProductGroup represents a group of products resulting from agriculture or livestock activities that vary only in certain well-described ways, being aggregated according to common biological traits. In this application, this field was instantiated with classes from APTO, which reuses some classes from AGROVOC and Agrotermos.
alm:productType	anyURI	Agricultural or livestock product type targeted by the commercial operation. In this application, this field was instantiated with classes from APTO, which reuses some classes from AGROVOC and Agrotermos.
alm:statisticalMethod	anyURI	Summary of the methods used for the process of obtaining data. Recommended best practice is to indicate the published resource URI in an open-access format.
alm:theme	Literal	Indicates the main subject or topic investigated in the economic statistical operation. Themes help categorize the dataset for easier discovery and analysis. The recommended best practice is to use a controlled vocabulary for consistency and accuracy. Examples of possible themes include Price Index, Domestic Material Consumption Indicator, Agricultural Production, Export and Import Data, Inflation Rate, Employment Statistics, and Energy Consumption.

## Footnotes

1 https://go-fair-agro.github.io/.

2 https://raw.githubusercontent.com/AlmesCore/APTO/refs/heads/main/apto.ttl.

3 https://w3id.org/APTO/.

4 https://agroportal.lirmm.fr/ontologies/APTO.

5 The metadata_id field was previously populated with strings for the IDs such as IpeaFumo, CepeaBoiGordo, ConabAcucarAM, etc.

6 https://data.pldn.nl/FilipiSoares/AgriPrices-1/.

7 https://api.data.pldn.nl/datasets/FilipiSoares/AgriPrices-1/sparql.

8 https://data.pldn.nl/FilipiSoares/-/queries/Query-1-1/9.

9 https://data.pldn.nl/FilipiSoares/-/queries/Query-2/1.

10 https://data.pldn.nl/FilipiSoares/-/queries/Query-3/1.

11 https://data.pldn.nl/FilipiSoares/-/queries/Query-3/2.

12 https://data.pldn.nl/FilipiSoares/-/queries/Query-4/1.
